# Applying fulvic acid for sediment metals remediation: Mechanism, factors, and prospect

**DOI:** 10.3389/fmicb.2022.1084097

**Published:** 2023-01-09

**Authors:** Chuxuan Song, Shiquan Sun, Jinting Wang, Yang Gao, Guanlong Yu, Yifu Li, Zhengqian Liu, Wei Zhang, Lean Zhou

**Affiliations:** ^1^School of Hydraulic and Environmental Engineering, Changsha University of Science and Technology, Changsha, China; ^2^Key Laboratory of Dongting Lake Aquatic Eco-Environmental Control and Restoration of Hunan Province, Changsha, China; ^3^School of Environmental Science and Engineering, Huazhong University of Science and Technology, Wuhan, China

**Keywords:** fulvic acid, molecular structure, physicochemical remediation, bioremediation, heavy metals, sediment

## Abstract

Fulvic acid (FA) has been shown to play a decisive role in controlling the environmental geochemical behavior of metals. As a green and natural microbial metabolite, FA is widely used in environmental remediation because of its good adsorption complexation and redox ability. This paper introduces the reaction mechanism and properties of FA with metals, and reviews the progress of research on the remediation of metal pollutant by FA through physicochemical remediation and bioremediation. FA can control the biotoxicity and migration ability of some metals, such as Pb, Cr, Hg, Cd, and As, through adsorption complexation and redox reactions. The concentration, molecular weight, and source are the main factors that determine the remediation ability of FA. In addition, the ambient pH, temperature, metal ion concentrations, and competing components in sediment environments have significant effects on the extent and rate of a reaction between metals and FA during the remediation process. Finally, we summarize the challenges that this promising environmental remediation tool may face. The research directions of FA in the field of metals ecological remediation are also prospected. This review can provide new ideas and directions for the research of remediation of metals contaminants in sediments.

**GRAPHICAL ABSTRACT:**
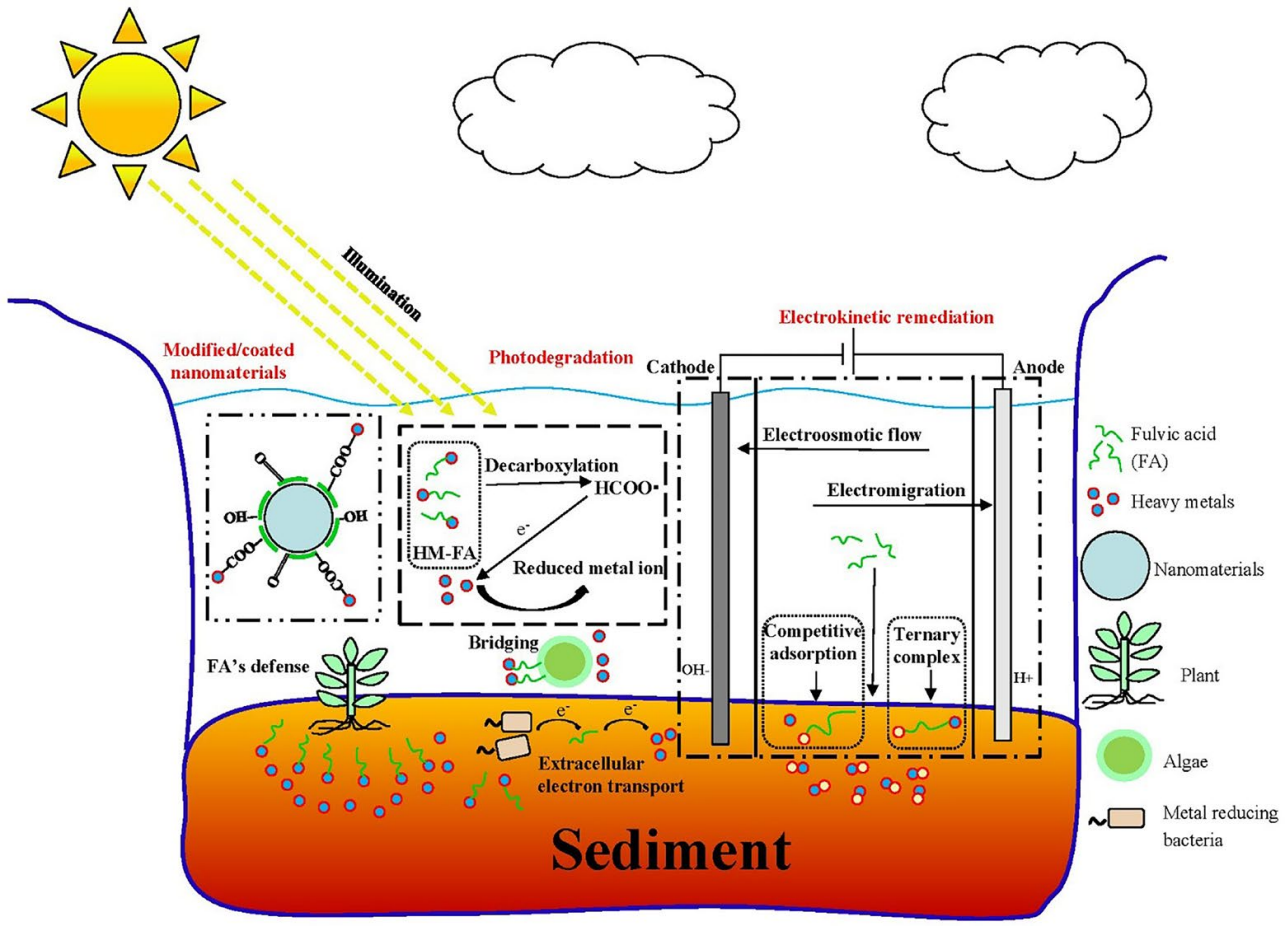

## 1. Introduction

Metal contamination has become a worldwide environmental problem because of the influence of fossil fuel burning, domestic sewage, industrial wastewater, municipal waste, chemical abuse, and other triggers (El Aafi et al., 2012; Kumar et al., 2017; Hanfi et al., 2019). Sediments are the final destination for most contaminants (Zhang M. et al., 2019; Song Z. et al., 2021). Metal contaminants in sediments have the characteristics of hard degradation, carcinogenicity, teratogenicity, mutagenicity, and biological amplification, which have significant impacts on ecosystems and human health (Monday et al., 2018; Tang et al., 2019; Jasu and Ray, 2021). Metals contaminants are widely distributed, and 43 important rivers in the world have varying degrees of metals contamination in their sediments. Common metal types include As, Hg, Cd, Cr, Cu, Zn, and Pb (Abhijit et al., 2021; Hossain et al., 2021; Oumayma et al., 2021). Metal pollution in the sediments of important domestic water bodies, such as the Yellow and Yangtze Rivers is becoming an increasingly serious problem (Yi et al., 2011; Weiqing et al., 2022; Xincheng et al., 2022). In Xiang River, the average metal concentrations of sediment samples from 22 typical metal-contaminated sections were much higher than the background values in 1982; specifically, the average concentrations of Cd, Hg, Zn, and Mn were 25.47, 7.40, 4.74, and 3.19 times the background values, respectively (Liu et al., 2017). Large amounts of metals stored in sediments pose extremely high ecological risk and threat to the health of the population (Hu et al., 2010; Sini et al., 2022; Yuefang et al., 2022). Long-term exposure of sediments to air leads to the oxidation of metal sulfide and release of toxic metals and high concentrations of acid to nearby water bodies (Anders et al., 2021). Moreover, sediments are resuspended when disturbed, releasing metal contaminants into the overlying water (Zhang Y. et al., 2020; Li et al., 2022). Therefore, the remediation of sediments contaminated by metals is imperative. In recent years, various physicochemical and biological methods for treating metal-polluted environment have been studied and developed (Lim et al., 2014). Many of the methods have yielded good results, but physicochemical remediation is costly, and prone to secondary contamination (Ali et al., 2013). Owing to the low bioavailability of metals and the long growth cycle of organisms, the large-scale application of bioremediation to practical remediation is still a challenge (Anekwe and Isa, 2022). Humic substances (HS) are potential remediation accelerators because they can significantly affect the environmental behavior of metal pollutants through complexation, ion exchange, and physical adsorption (Baohua et al., 2011; Piccolo et al., 2019; Guangren et al., 2022; Zhe et al., 2022).

Fulvic acid (FA), the main component of the widely distributed HS in the environment, plays a dominant role in governing the environmental behavior of metals (Matilainen et al., 2011; Morales et al., 2012; Qianting et al., 2022). In a water environment, FA can precipitate metal contaminants after adsorption, or FA-modified materials are used to adsorb contaminants (Li et al., 2017; Wang H. et al., 2021). In the soil environment, FA can reduce the mobility of contaminants by adsorbing metal contaminants and binding to soil minerals or to promote plant growth (Dos Santos et al., 2020; Peng et al., 2022). As an environmental medium between soil and water, sediments are excellent arenas for FA to play a role in metal remediation. The carboxyl and phenolic groups of HS are mainly involved in the complexation of metals ions (Li et al., 2018). As a promising metal remediation tool, FA has more oxygen-containing functional groups (such as carboxyl, phenolic hydroxyl, alcoholic hydroxyl, amino, quinone, sulfhydryl, and methoxy) than humic acid (HA) and humin. Therefore, it binds metal ions more readily than other components of HS and has a significant impact on the migration, transformation, toxicity, and bioavailability of metals in sediments (Donisa et al., 2003; Xu et al., 2019; Bondareva and Fedorova, 2020). Compared with other artificial chemical additives, such as acetylacetone, anthraquinone-2, 6-disulfonate, FA has the advantages of being green and low cost (Peng et al., 2022). Moreover, FA can act as a reducing or oxidizing agent to participate in the redox reactions of metals contaminants, thus having a significant impact on ecological restoration (Watanabe et al., 2005). Based on its molecular structure properties and redox activity, FA can form a complex with nanomaterials to adsorb or passivate metal pollutants, can be used as a photosensitizer to improve photodegradation rate (PD), and can be used as a chelating agent to improve the efficiency of electrokinetic remediation (EKR; Wang H. et al., 2021). In bioremediation, FA can be used as an adsorbent, electronic shuttle agent, and biological accelerator to improve the speed and effect of bioremediation on metal contaminants (Calza et al., 2014; Yang et al., 2018; Minfen et al., 2022). In recent years, FA has attracted considerable interest (as shown in Supplementary Figure 1). To date, FA is widely used in soil remediation, but its application to sediment remediation has not been systematically reviewed. Therefore, summarizing the interaction between FA and metals in sediment environment and the application of FA to remediation technology is necessary.

Fulvic acid can affect the migration, adsorption, complexation, and fugacity patterns of metal contaminants. Naturally, FA has a non-negligible influence on metal adsorption, photodegradation, migration, transformation, and biodegradation. However, no systematic review on the effect of FA on metal contaminant remediation has been conducted. Thus, it is of considerable practical interest to reveal the engineering application potential of FA as a metal remediation agent. In this review, we revealed the binding characteristics of FA and metals and the application mechanisms in remediation. Meanwhile, we summarized the latest progress in the use of FA in the remediation of metal pollutants, focusing on the impact of FA on physicochemical and biological remediation. The factors, phenomena, and challenges involved in the process are also discussed. Finally, future perspectives are offered to stimulate more excellent enhancements in this promising field. This article will provide help and reference for promoting the further application of FA in the field of metals remediation.

## 2. Mechanism of FA controlling metal environmental behavior

The basic mechanism by which FA controls the environmental behavior of metals is based on the molecular structural features of FA. It contains many functional groups, such as aliphatic, hydroxyl, amide, quinone, ketone, and carbonyl, which can react with metals (Lu et al., 2000; Aranganathan et al., 2020; Arti and Ritika, 2021). Li et al. (2018) found that nitrogen-and oxygen-containing groups play a major role in the adsorption of metals, mainly in strong binding sites (nitrogen-containing groups) and weak binding sites (phenol groups and carboxyl groups). Electrostatic attraction plays a dominant role in the adsorption process. Negatively charged functional groups after deprotonation can attract metal cations. In redox interactions involving FA, the quinone groups of HS are the key functional groups in HS redox activity (Peng et al., 2022). These groups cycle between oxidized semi-quinone radicals and hydroquinone through redox processes. The possible three-dimensional diagram of an FA is shown in Supplementary Figure 2. This unique molecular structure of FA results in a large cation exchange capacity and redox ability. Compared with other dissolved organic matter (DOM), FA can form stronger bonds with metals ions (e.g., Cd and Cr; Gillispie et al., 2016; Isrun et al., 2021). The adsorption capacity of FA on metals can reach a value about 20 times that of HA (Borůvka and Drábek, 2004; Xiaoqing et al., 2022). Zhang Z. et al. (2020) have comprehensively explained the binding mechanism of FA and metals by combining experiments, models, and component characterization; their research shows that the proportion and molecular weight of oxygen-containing functional groups containing FA are the dominant factors for the binding between FA and metal ions, consistent with previous conclusions (Jeong et al., 2007; Boguta et al., 2016; Zhang et al., 2018). Maksym et al. (2021) confirmed that quinone carbon–oxygen double bond, phenol hydroxyl, and carboxyl sites are the most reactive in a class of FA molecule during the reactions with metal cations and can form stable complexes with Cd (II), Cu (II), Mg (II), Ni (II), Pb (II), and Zn (II). These studies have indicated that the adsorption and passivation of FA on metals are mainly related to the molecular weight and the abundance of oxygen containing functional groups of FA.

The factors that affect the adsorption, migration, and biological toxicity of FA to metals have gradually emerged. The adsorption capacity of FA varies by metal, showing a general trend Cu^2+^ > Pb^2+^ > Cd^2+^. This phenomenon may be mainly due to the ionic and hydration radii of metal ions (Li et al., 2018). Notably, different sources of FA exert varying effects on metals, for example, FA extracted from the soils of the Amazon region showed more metal ion complex sites than FA extracted from other regions. The difference was mainly attributed to the molecular structure and functional group content of FA (Dos Santos et al., 2020). This finding shows that the source of FA and metal type are the key factors affecting interactions between FA and metals. Moreover, significant differences in the effects of FA on metals have been found among different environmental media. Kouhail et al. (2020) evaluated the influence of FA and HA on the migration of indium (Yn) and gallium (Ga) in quartz sand through a sand column experiment; the presence of FA at a certain concentration enhanced the fluidity of Yn and Ga in a quartz sand medium; the mobility of FA to Yn and Ga increased by 18 and 34%, respectively, relative to that of DOM, but the influence of FA on the fluidity of Yn and Ga was smaller than that of HA; this phenomenon may be due to the low fat/aromatic ratio in the functional group of FA and consequent inhibited adherence of FA to the mineral surface. This result is consistent with previous research results. The elution effect of FA on As, Ca, and Fe is significantly better than that of HA (Sun et al., 2022), indicating that the adsorption of metals is stronger in the solid phase by FA than by HA. Apart from that, in a microplastic environment, the adsorption capacities of microplastics with different aging degrees to Pb^2+^ increase with FA content (Guan et al., 2022). In addition, HS exhibited different metal binding abilities in acidic and alkaline environments. FA and HA in Pony Lake had strong Ag^+^ binding capacity, whereas HA and FA in Suwannee River had weak Ag^+^ binding capacity. These findings confirm that pH value can affect the degree of binding between Ag^+^ and HS to some extent (Mousavi et al., 2015). Xun et al. (2022) showed that this combination facilitates the adsorption of FA molecules on the surface of silver nanoparticles (AgNPs) in water, and FA molecules increase the particle sizes and negative charge of AgNPs, decreasing the amount of dissolved AgNPs and indirectly protecting fish from toxicity. However, in a river sediment environment, FA may increase the accumulation of AgNPs in organisms, such as nematodes and *Escherichia coli*, and may thereby increase the potential risk of AgNPs to human health through the food chain (Luo et al., 2022). Under acidic conditions, FA increases the oxidation rate of molecular oxygen to Fe (II); this effect indicates that FA affects the biological toxicity of metal contaminants through redox reactions in natural environments (Jones et al., 2015; Peng et al., 2022). In conclusion, the adsorption complexation capacity and redox ability of FA are mainly related to its molecular structure, molecular weight, and source. In addition, pH and competing components can significantly alter the effect of FA on the migration capacity and biotoxicity of metals.

**Figure 1:**
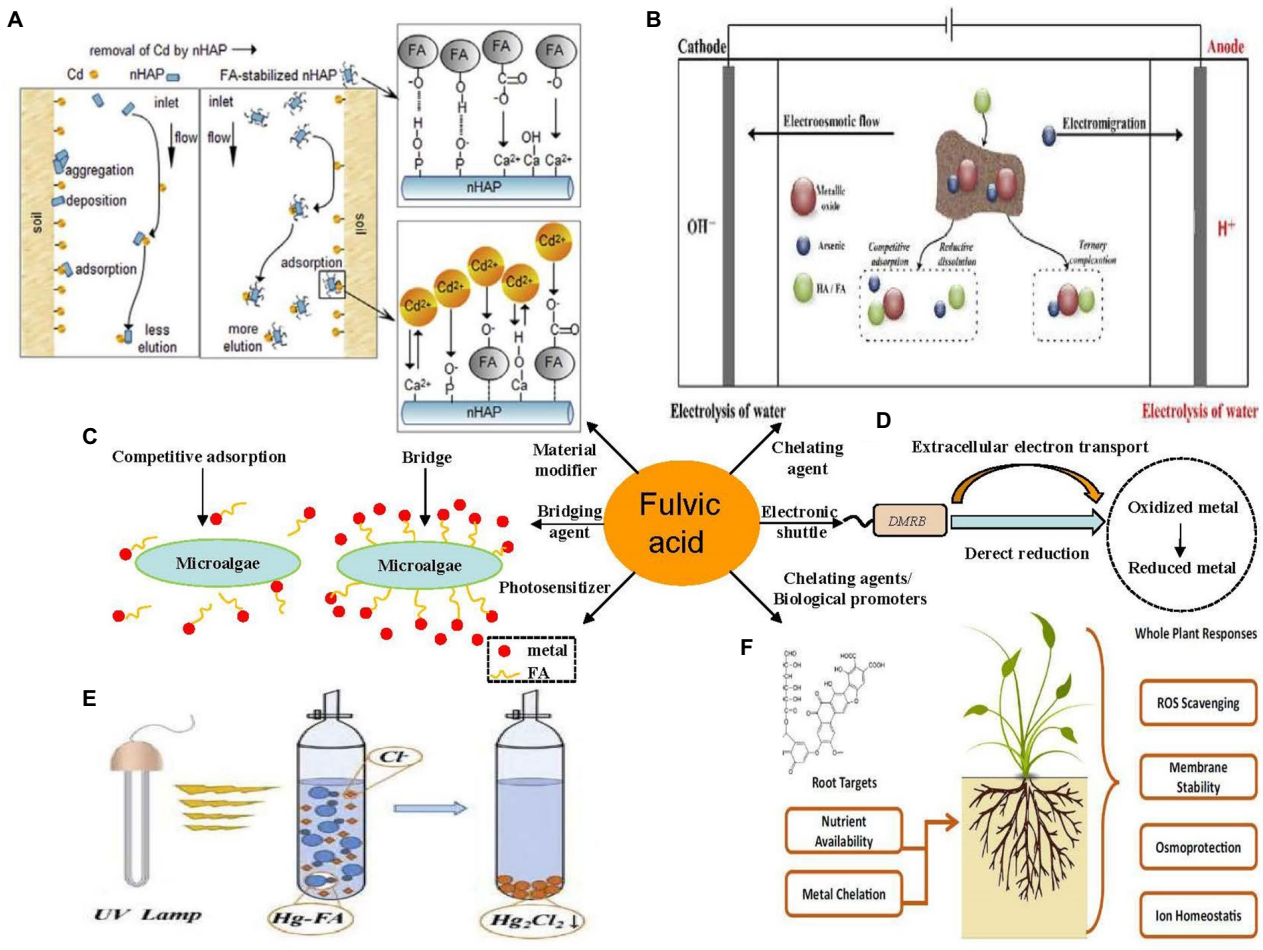
Main role of fulvic acid (FA) in physicochemical and bioremediation of metal contaminants. **(A)** Modification of nanomaterials (Li et al., 2019). **(B)** Electrokinetic remediation (Li J. et al., 2020). **(C)** Photodegradation (Fan et al., 2018). **(D)** Microalgal remediation. **(E)** Microbial remediation. **(F)** Phytoremediation (Van Oosten et al., 2017). All panels were obtained with the permission from publishers.

The primary mechanism of FA-assisted metal contaminant remediation is shown in Figure 1. FA can composite with nanomaterials to form new materials and results in the strong adsorption of metals in the environment. This is because surface bound FA can introduce oxygen-containing functional groups and negative charges into nanomaterials, thus increasing the apparent adsorption of metal ions through chemical complexation and electrostatic attraction, respectively (Tang et al., 2014). Moreover, it can reduce the agglomeration of nanomaterials and promote the reactivity of nanomaterials and metals. During EKR, FA promotes the EKR effect in three ways: (a) FA occupies the adsorption sites on the solid phase through competitive adsorption to promote the release of metals; (b) FA promotes metal release by reducing dissolved metal minerals; and (c) FA formed a ternary complex with metal ions to promote the release of metals in the environment (Li J. et al., 2020). A large number of radical components (e.g., quinone or semi-quinone) in natural DOM act as electron transfer mediators that accelerate the photoreduction of metals in the oxidized state (Berkovic et al., 2012). Under light (especially under UV radiation in the range of 290–400 nm), the type and abundance of an FA functional group, molecular structure, chemical composition, configuration, and conformation undergo significant changes (Cho and Choi, 2002; Filipe et al., 2020; Sun et al., 2022), and thus FA can mediate the migration, transformation, and other environmental behavior of metals in sediments. For example, FA–Hg (II) complexes are decomposed into many small molecular substances after UV light treatment, and a large number of highly reducing substances (e.g., COO and –COOH), which can mediate the reduction of Hg (II), are produced in this process. FA may produce hydroxyl and superoxide radicals (^1^O2) under UV-A radiation and thereby increase the PD rate of MeHg (Kim et al., 2018). The effect of FA on microorganisms (microalgae, bacteria, and fungi) and metal contaminants in a sediment environment is complex. As a microbial metabolite with good affinity for metals, FA can reduce the bioavailability of metals (Hongyang et al., 2022) and can form multiple complexes with metals and microorganisms to stabilize (Shi et al., 2017; Kulkarni et al., 2018). As an electron shuttle and gene expression regulator, FA can facilitate extracellular electron transfer in bacteria and can act as an adsorption bridge between microalgae and metals ions (Zhao et al., 2015). In the phytoremediation process, FA mainly improves the tolerance of plants to metal pollutants through its metal-chelating capacity and promotes plant growth by regulating plant metabolism (Van Oosten et al., 2017; Qiu et al., 2021). As a compound with similar molecular structures and behavior characteristics, FA has great application potential in the remediation of metal-contaminated sediments.

## 3. Role of FA in the remediation of metals contaminants

### 3.1. Physicochemical remediation

Physicochemical remediation technology mainly uses physical methods to separate and immobilize metals, coupled with chemical methods to reduce their content. Physicochemical remediation has the advantages of simple operation and fast results, but the cost and secondary contamination problems have hindered its development (Song Y. et al., 2021). Natural FA is an important component of DOM and exhibits high complexing ability and redox activity. Therefore, FA as an agent to enhance the physicochemical remediation of metals pollutants has attracted considerable interest. In the physicochemical remediation of metal contaminants, nanomaterials, photodegradation techniques, and electrochemical techniques in the remediation of metals contaminants have been widely studied. Hence, we will focus on the role of FA in these areas. Table 1 shows that FA can generally enhance physicochemical remediation effect several times. In this section, the great potential of FA in this field will be revealed.

**Table 1:** Impact of FA on physicochemical remediation for metals contaminants.

Remediation technology	Advantages	Limitation	Strategies	Metals	Effects	Removal rate	Cites
Nanomaterials remediation	Large surface area, high reactivity	Secondary pollution, cost	Elevate the stability, and reactivity of nZVI	Cr(VI)	At pH = 9 and C_FA_ = 10 mg/L, the Cr^6+^ removal rate improved by 35%.	60%	Dong et al. (2016)
			Enhance migration of nHAP	Cd	Elution rate of soil Cd increased approximately 13.2-fold (from 0.64 to 8.46%) compared to the control group; In the FA concentration range of 20–500 mg/L, the removal rate increased linearly with increasing promoter concentration	8.46%	Li et al. (2019)
			Coated nanosilica	Pb(II), Cu(II), and Zn(II)	At pH = 5, the adsorption capacities of Pb, Cu, and Zn were about 2.2, 2.3, and 2.7 times higher than those of the control group, respectively	55–60%	Liang et al. (2011b)
			Synthesis of Fe_3_O_4_/MnO_2_/FA nanocomposites	Sr(II)	The adsorption capacity of Sr^2+^ ions at 25°C and pH = 8 is 227.3 mg/g	100%	Ghaeni et al. (2019)
Electrokinetic remediation	High efficiency, simple process;	Large energy consumption, high cost, and secondary pollution	Leach of metals as adsorbents and chelating agents	Cd, Co, Mn, Ni, Pb, and Zn	About 1.0–2.0-fold increase in removal efficiency of metals by EKR compared to unenhanced. The EKR effect is related to the distance from electrode.	Cd:55–80%	Bahemmat et al. (2016)
						Co:75–100%	
						Mn:65–100%	
						Ni:60–95%	
						Pb:35–75%	
						Zn:30–95%	
				As	About 2.8-fold increase in removal efficiency of metals by EKR compared to control experimental group	39%	Li J. et al. (2020)
Photodegradation remediation	Low cost	Secondary pollution, light intensity	As photosensitizer and electron	Hg(II)	About 70% removal rate of mercury for real industrial wastewater The mercury removal rate of the simulated wastewater is about 90%, which is about 16 times that of the control group	70–90%	Fan et al. (2018)
			As photosensitizers	Methyl-mercury (MeHg)	PD rate reached about 0.67, an improvement of 280.95% compared to the control group	67%	Kim et al. (2018)

#### 3.1.1. Modified nanomaterials remediation

Environmental remediation technologies for modified nanomaterials are based on the ability of certain materials or modifiers to adsorb or passivate metal contaminants. A large number of new efficient and economical DOM-modified nanomaterials have been developed and widely used in environmental remediation (Dutta et al., 2022; Pratyush et al., 2022; Rajput et al., 2022). The role of HS in changing the surface properties, transport capacity, aggregation, and toxicity of nanomaterials has drawn the attention of researchers (Khin et al., 2012; Tang et al., 2014). More FA was adsorbed on the nZVI surface at pH values 5–7 than at pH 9, and thus the effects of FA on the sedimentation performance of nZVI varied at different pH values. The settling performance of nZVI particles at pH 7 was significantly lower than that at pH 9 in the presence of the same concentration of FA. Moreover, Cr(VI) removal rate improved by about 35% at pH 9 and C_FA_ = 10 mg/L. However, at the same FA concentration, FA showed obvious inhibition on the reduction reaction at pH 5, whereas at pH = 7, only FA at low concentration (C_FA_ = 2 mg/L) promoted the reduction reaction of Cr (Dong et al., 2016). This result showed significant change in the kinetics of metals removed by nZVI in the presence of HS (Mak and Lo, 2011). The colloidal stability and reactivity of nZVI change in the presence of FA. When FA content is extremely high to occupy the active surface position of nZVI, the reduction effect of nZVI on Cr(VI) is reduced even if FA enhances the dispersion of nZVI particles. When FA content is low, the adsorption of FA on the nZVI surface can enhance the stability of particles and promote the reduction of Cr(VI) by providing more available surface positions (Dong et al., 2016). Liang et al. (2011a,b) compared the ability of SiO_2_ nanoparticles (20 nm) to adsorb Cu (II), Pb (II), and Zn (II) metal ions before and after coating FA on the surface; the results showed that the adsorption ability of nano-SiO_2_ on metal ions was significantly enhanced after coating with FA. Owing to the unique crystal and surface structure of nano-hydroxy (nHAP), how to use modified nHAP in the remediation of metal contaminants in the environment has become a major subject of interest worldwide in recent years (Katoh and Hamada, 2021; Ahmed et al., 2022; López et al., 2022). Li et al. (2019) studied the effect of nHAP suspension at different concentrations of FA on the elution of Cd from the soil; the results showed that the elution rate of Cd from soil increased by 2.78, 3.31, 4.37, 5.45, and 8.46% after FA of 20, 100, 200, and 500 mg/L was added to nHAP nanofluids, showing that FA effectively facilitated Cd removal by nHAP in soil. Researchers studied the binding mechanism of nHAP and Cd (II) affected by FA and montmorillonite; the results showed that FA increased the electronegativity of nHAP by adsorbing carboxyl and phenolic groups on the surface of nHAP particles, forming a space resistance effect, which prevented the reduction of the specific surface area of nHAP particles and increased the adsorption of nHAP on Cd (II). In a subsequent study, the experimental data showed that FA increased the mobility of nHAP by 58–75% and FA improved the stability of nHAP particles, enhanced the electrostatic repulsion between nHAP particles and sand, and reduced the retarding effect of nHAP on Cd (II) migration (Wu et al., 2020; Mengmeng et al., 2022).

Research on FA-assisted other nanocomposites is continuous. Ghaeni et al. (2019) prepared reusable and low-cost Fe_3_O_4_/MnO_2_/FA nanocomposites that have high adsorption capacities and high desorption efficiency and can be used to extract Sr (II) and Ca (II) from the environment. Gel nanocomposite FA-based surface coated crumbs (GN/fabc) provide a new method for the treatment of bread waste and the efficient utilization of resources while effectively removing Pb (II) from water. A pot experiment showed that GN/fabc inhibits the bioavailability of Pb (II) and this material shows great potential for bioremediation technology coupling (Cai et al., 2022).

Although many modified nanometer materials have been used with some success in the remediation of metals contaminants in sediments, the potential of the materials for secondary contamination, the cost of practical applications, and the potential for coupling with other material remediation technologies have not been thoroughly explored. Therefore, improving the recyclability of nanomaterials (such as doping magnetic materials) and changing the surface hydrophobicity of nanomaterials (such as modifying with FA and citric acid) will be the next research focus in this field (Tang et al., 2014). In addition, using nanomaterials to support microorganisms (chlorella and metal-reducing bacteria) is also a research hotspot (Shiying et al., 2017; Zhao et al., 2022), and FA may emerge as a binder in this field.

#### 3.1.2. Electrokinetic remediation

Electrokinetic remediation, which emerged in 1993 (Acar and Alshawabkeh, 2002), is widely used in the remediation of metals contaminants because of its advantages of high energy efficiency, easy operation, and low cost (Huichao et al., 2021; Miaomiao et al., 2022; Yunfeng et al., 2022). Many chelating agents that contribute to EKR technology have been developed, but these reinforcers often cause secondary contamination to the environment (Baek et al., 2008; Isosaari and Sillanpää, 2012; Arulpoomalai and Shashidhar, 2021; Sadat et al., 2021).

The influence of chelating agents on EKR technology has been developed, but these reinforcers often cause secondary pollution to the environment (Bulman et al., 1998; Krachler et al., 2015). Bahemmat et al. (2016) confirmed that FA can greatly enhance the efficiency of EKR in metal-contaminated soil remediation; after 25 days of experiments, the removal efficiency of As in soil enhanced by FA is two or three times that of nonenhanced EKR. However, FA at a high concentration may not effectively promote EKR because it may reduce soil porosity and cause soil hardening, which reduce the electrical conductivity and significantly reduce the EKR efficiency of metals (Krachler et al., 2015). FA at an appropriate concentration can promote the release of metals in soil through competitive adsorption, reductive dissolution, and complexation and improve the EKR efficiency of metal pollutants. In this process, FA showed a better enhancing effect than HA (Güngör and Bekbölet, 2010; Li J. et al., 2020).

Fulvic acid can form complexes with metals and enhance metal transport under an electric field. However, excess FA is ineffective in enhancing EKR possibly because of severe changes in pH, which affect the zeta potential and may reverse the direction of the electroosmotic flow during metal electromigration (Wang Y. et al., 2021). If ionic contaminants migrate to the direction opposite to the electroosmotic flow, removal is prevented (Cameselle, 2015). FA is a precursor of disinfection by-products, so the use of FA needs to be strictly controlled in practical applications (Zhang and Yang, 2018). In the application of FA-assisted sediment EKR, the concentration and dosage of FA should be fully controlled.

#### 3.1.3. Photodegradation remediation

As a widely recognized excellent photosensitizer, HS has an important impact on the photodegradation of contaminants in water and sediments (Zhou et al., 2020; Meiying et al., 2022). FA at an appropriate concentration can be used as a photosensitizer to enhance the photolysis rate of contaminants, such as polycyclic aromatic hydrocarbons, algal toxins, and antibiotics (emerging fluoroquinolones and sulfamethazine; Xia et al., 2008; Ren et al., 2019; Zhuojuan et al., 2022). However, studies on the effect of FA on the photodegradation remediation of metal contaminants are still in infancy. Fan et al. (2018) showed that FA can reduce Hg(II) to Hg_2_Cl_2_ (a slightly toxic precipitate) under UV irradiation, thereby significantly reducing the biological toxicity of Hg; in UV treatment without FA, the degradation of Hg(II) was minimal, and the removal efficiency was only 3.9%; however, when FA was added to the system (mass ratio of FA to Hg^2+^ ion was 2:1), the photodegradation efficiency of Hg(II) significantly increased from 8.6 to 60.8%; this method effectively recovered Hg from high-concentration Hg(II) acidic wastewater, exhibiting good commercial application value. Kim et al. (2018) investigated the influence of DOM, such as FA, on the PD of methyl Hg in artificial estuarine water under UV-A radiation; the data showed that FA in estuarine water significantly increased the PD of methylmercury (MeHg) in artificial simulated seawater; however, the effect of photodegradation on the biotoxicity of some metal ions may be negative. Liu et al. (2022) found that FA-metal ion complexes are photodegraded under the vacuum ultraviolet/hydrogen peroxide (VUV/H_2_O_2_) process, which in turn explains the release of free state ions (Pb^2+^, Cd^2+^) into the water column. In summary, FA may be more suitable to the remediation of metals, such as Hg, Cr, U, and As, whose toxicity is strongly influenced by valence state (Chen et al., 2022).

Many similar studies have emphasized that sunlight is critical for the photodegradation of metal contaminants, but sediments in natural water bodies often lack light. Therefore, in the practical applications of photodegradation remediation, FA may be more suitable for the *ex situ* remediation of sediments, such as dredged sediments. In addition, the pH value of a system and the aromaticity and humus content of DOM decrease with increasing duration of light exposure; that is, the concentration of FA is difficult to maintain at an appropriate level. After photodegradation, the ability of DOM to bind Cu^2+^ and Zn^2+^ in aqueous solutions is significantly reduced, and this effect may lead to the release of metals originally bound to DOM. Hence, the use of FA-assisted photodegradation requires the strict control of light intensity and duration to light exposure, and the environmental impact of photodegradation on the ability of natural DOM to adsorb metals ions should be evaluated in advance according to the type of pollutants and the pollution level in sediments (Yin et al., 2019; He et al., 2021).

### 3.2. Bioremediation

Bioremediation is a green and economic remediation technology for metal-contaminated sediments. Compared with physicochemical remediation methods, it has more environmental benefits. Bioremediation mainly uses microorganisms (microalgae, bacteria, and fungi), plants, and other natural methods to alleviate metal-contaminated sites. It is less harmful to the environment but has problems, such as slow biological growth and great seasonal impact. FA exerts effects that greatly promote biological processes (Lin et al., 2022). Its role in bioremediation is shown in Table 2.

**Table 2:** Impact of FA on bioremediation for metals contaminants.

Remediation technology	Advantages	Limitation	Strategies	Metals	Effects	Removal rate	Cites
Microbial remediation	Low cost, simple operation	Competition of indigenous bacteria	Use FA as electronic shuttle, and complexing agent	Cr(VI)	The reduction of Cr(VI) by DOM-promoted bacteria is about doubled (from 29% to about 57%)	57%	Dou et al. (2022)
			Use FA as electronic shuttle and gene regulator	Fe(III)	The degradation rate of Fe (III) by bacteria in FA batch is 66% higher than that in control batch	53%	Wang et al. (2020)
			Use FA to promote the growth of fungi, and reduce the toxicity of metals	Mn, Pb	Low concentration of metals reduces the biological toxicity to fungi	Not mentioned	Malcová et al. (2002)
Phyto-remediation	Good remediation effect, green	Long growth cycle, seasons	Use of FA as leaching agent and microbial promoter	As, Fe, and Mn	The contents of As, Fe, and Mn in soil pore water increased by 12, 20, and 3 times, respectively.	Not mentioned	Yi et al. (2019)
			Use FA as leaching agent of mineral mixture	Fe	The iron concentration in pore water is about seven times that without FA.	Not mentioned	Cerozi (2020)
			Use FA as a bio-promoter	Cd	Facilitate Cd accumulation in microalgae (about 2.8 times more than the control).	44%	Gao et al. (2022)
			Strengthen the adsorption of metals by microalgae, and reduce the biological toxicity of metals through competitive adsorption, and bridge action	Ni, Zn	The maximum concentrations of microalgae to Ni and Zn were 0.26 and 0.68 mmol/L, respectively, which are higher than those without FA	Not mentioned	Zhang et al. (2022)
				Cu	The maximum allowable concentration of microalgae to Cu was about 2.4 and 4.4 times higher than that of the control	Not mentioned	Shi et al. (2017)

#### 3.2.1. Microbial remediation

Microorganisms are widely distributed, small in size, fast in reproduction, and easy to cultivate. Bacteria in microorganisms are widely used to remediate metals in the environment, and many kinds of bacteria can degrade and adsorb metals on a large scale without any human interference (Naz et al., 2016). For example, researchers have identified a new Hg-resistant strain for the microbial remediation of Hg-contaminated sediments (Mahbub et al., 2016). A novel biofilm with good biosorption capacity for Pb contaminants was extracted from sediments of the Sano estuary, which can serve as a good tool for Pb contamination removal (Pepi et al., 2016). Many native bacteria can control the toxicity of metals by altering the form in which they occur and affect the toxicity and transfer of metals (especially Hg, Cr, etc.; Sarwar et al., 2017). In recent years, many impressive achievements have been made in the artificial control of microbial remediation, including biological stimulation and bioaugmentation technology (Li et al., 2016; Samuel et al., 2021). As a good extracellular electron shuttle and complexing agent, FA facilitates the microbial remediation of metals (Yong et al., 2016; Rosenmai et al., 2018). As shown in Table 2, the use of FA to promote the remediation of metal-contaminated sediments by bacteria and fungi is effective.

**Figure 2:**
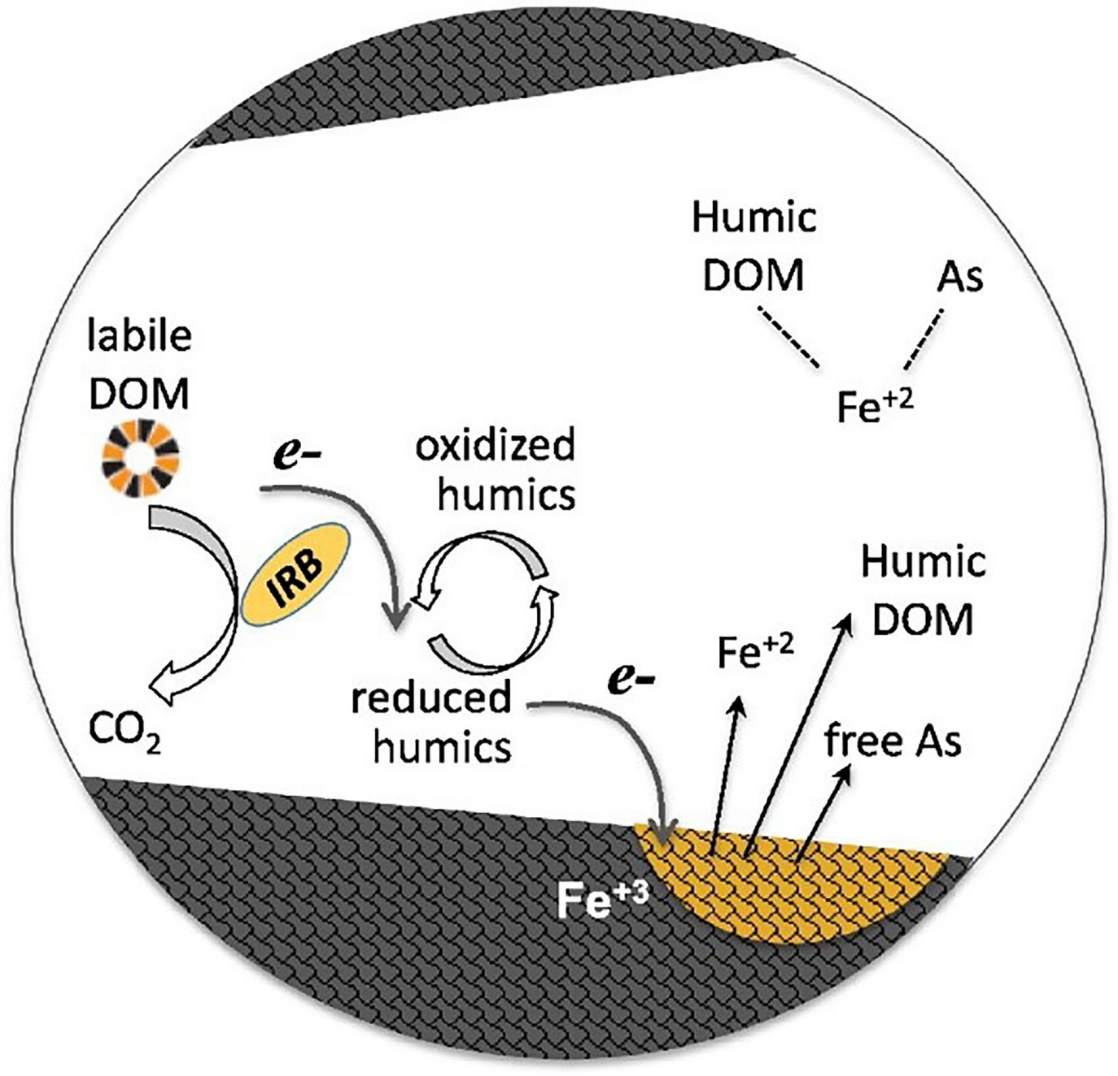
Conceptual model of humic dissolved organic matter (DOM) as an electronic shuttle that enhances the reductive dissolution of Fe(oxygen) hydroxide minerals (Kulkarni et al., 2018). Panels were obtained with the permission from publishers.

Many studies have found that HS can effectively mediate the extracellular electron shuttle of dissimilatory metal-reducing bacteria (DMRB) and promotes intermediate collaboration and oxidation/reduction of metals in bacteria (Dang et al., 2016; Zhang L. et al., 2020). The efficiency of Cr(VI) reduction by *Geobacter sulfurreducens* (a common DMRB in anaerobic soils) is positively correlated with the HS content of DOM in the environment. The amount of Cr (VI) reduced by DOM-promoted sulfur reducing bacteria roughly doubled (from 29 to 57%; Dou et al., 2022). Furthermore, Kulkarni et al. (2018) demonstrated that FA can shuttle electrons between *Geobacterium* metal-reducing bacteria and Fe(III) and promote the reduction of ferric iron; the generated free Fe (II) complexed with other molecules (such as humic DOM and ternary complexes of As and Fe). Its conceptual model is shown in Figure 2. A study showed that FA can effectively promote and mediate the reduction of Fe(III); after 2 days of the experiment, the concentration of Fe(III) in the FA batch was 66% higher than that in the control batch, and the accumulation of Fe(II) content in the FA batch was significant, reaching 152.4% of the control batch (6.60 and 4.33 mM, respectively). FA not only mediated extracellular electron transfer but also significantly regulated the expression of genes related to cation binding, metal ion binding, flagellation, and electron transfer activity of *Geobacter sulfurreducens*, which plays a key role in Fe(III) reduction. In addition, trace amounts of FA promoted Fe(III) reduction and Fe(III) ion binding by regulating the expression of Fe and FeS transporter genes (Wang et al., 2020). FA plays a key role in controlling the bioavailability of Hg in plateau wetlands. In the sediments of seasonally flooded areas, FA can greatly increase the production of methyl Hg in sediments and the bioaccumulation of microorganisms. However, FA tends to retain Hg in permanently flooded sediments, and this effect is not conducive to the bioavailability of metals. FA with different molecular composition and sources has obvious spatial heterogeneity in terms of the effects of methyl mercury and microorganisms in wetland sediments (Xu et al., 2021). Overall, FA can be used as a good extracellular electron shuttle and a regulator of gene expression.

Fungi with good metal uptake and recycling abilities are widely used to remove metals from the environment, such as *Aspergillus flavus, Aspergillus niger, Aspergillus oryzae*, and halophilic fungi (Bano et al., 2018; Igiri et al., 2018). Malcová et al. investigated the effect of FA on the growth of the fungus *Pseudomycetes* and the toxicity of Pb and Mn; the results showed that a certain concentration of FA (27.1 mgC/L) promoted fungal proliferation. The toxicity of metals to fungi was reduced when FA (81.3 mgC/L) was added to solutions containing certain concentrations (less than or equal to 0.1 mmol/L) of Mn and Pb; however, the positive effect of FA was not evident at higher concentrations of metals (0.5 mmol/L; Malcová et al., 2002). Recent studies have found that the addition of FA can affect the environmental ecological niche, improving the alkalinity, nutrient status, and microbial activity of the soil environment and thus optimizing the structure of the fungal community in the environment (Jin et al., 2022). In conclusion, FA can be used to promote fungal proliferation and optimize microbial structure.

Fermentation composting technology has attracted considerable interest as an efficient and convenient technology for combined microbial remediation and resource recovery. HS in compost products can be used as a good detergent to effectively remove Cd and Ni from sediments (removal rates of more than 70 and 40%, respectively; Zhang S. et al., 2019). FA can improve microbial structure in the composting process and thus improve the metal remediation effect. Li et al. found that adding FA at appropriate concentration in the composting process with rice straw and mushroom residue as the substrate can improve composting quality. Most microbiota (nearly 80%) have significant reactions to FA during composting. The abundance of pathogenic bacteria, such as *Corynebacterium*, *Elizabeth kings*, and *Sarcocystidae*, decreases, whereas the abundance of beneficial microorganisms, such as *Aeribacillus*, *Oceanobacillus* and *Rhodospillaceae*, increases significantly. In addition, FA can reduce ammonia volatilization during composting, improve nitrogen source utilization efficiency at the initial stage, and accelerate heating rate. Finally, compost products have rich nutrition and good biological characteristics (Li et al., 2021; Yao et al., 2021). In conclusion, FA is a powerful tool to promote the passivation of metals during composting.

Microbial remediation as an *in situ* or *ex situ* remediation technology for remediating metals in sediments. FA has significant potential to mediate microbial remediation of metals contaminants in sediment environments, but high concentrations of FA may have toxic effects on microorganisms, and FA at a low concentration may be an ineffective electron shuttle and gene regulator. Therefore, controlling the concentration, molecular weight, and source of FA is the key to the application of FA to actual microbial remediation projects.

#### 3.2.2. Phytoremediation

Phytoremediation technology mainly uses the adsorption, accumulation, degradation, volatilization, and other effects of various plants on metals (He and Chi, 2016; Kurade et al., 2021; Lijun et al., 2021). Many plants have strong metal tolerance by combining metals ions with the cell wall and actively transporting them to vacuoles to reduce metals stress (Memon and Schröder, 2009; Esringü et al., 2021; Chamika et al., 2022). In recent years, many physicochemical remediation technologies have been developed for combined remediation (Xiaomin et al., 2021; Gu et al., 2022). They have achieved certain results but are expensive or easily cause secondary contamination. DOM has been used to improve the efficiency of metal phytoremediation (Borggaard et al., 2019; Min et al., 2021). As an important component of DOM, FA can act as a chelating agent and facilitator to enhance plant activity or promote interspecific collaboration between plants and microorganisms. The interaction between rhizosphere microorganisms and plants plays an important role in metals biogeochemical cycle (Guo et al., 2019; Zhang H. et al., 2020; Rane et al., 2022). Some rhizosphere microorganisms are resistant to metals and can promote plant growth (Wiangkham and Prapagdee, 2018). The main reason is that some metabolites of these rhizobacteria can decrease the bioavailability of metals in sediments (Teixeira et al., 2014; Li Y. et al., 2020).

Fulvic acid can compete for the adsorption of metal contaminants in the environment, thus reducing the effect of metals on plants. Huang et al. (2020) found that FA and HA can reduce the toxicity of Ag ions in rice owing to reduction in silver ion concentration. FA can act as a bio-promoter to directly promote plant growth. Gao et al. (2022) confirmed that HA and FA can be used as biological promoters to significantly increase the accumulation of Cd in *Sedum Alfredia* (2.17 and 2.78 times that of the blank control group, respectively). In addition, hydroponic plants usually lack iron, and FA is a chelating agent to increase available iron and reduce iron pollution (Cerozi, 2020). Finally, FA can indirectly assist phytoremediation by promoting the growth of root-based microorganisms. Ran et al. (2022) conducted an in-depth study on the effects of FA on Hg methylation and bioaccumulation in paddy soil; the results showed that the addition of FA greatly increased the abundance of Hg methylation microorganisms and low-molecular-weight organics (such as cysteine) in paddy soil, thereby improving the mobility and methylation of Hg in soil and increasing the absorption of metals by plant roots. Yi et al. (2019) found that the addition of 0.2–1.5% FA had a significant impact on the mobility of As and the composition of the microbial community in paddy soil. The concentrations of Mn, As, and Fe in soil pore water were 3, 12, and 20 times those in the control group, respectively. Moreover, FA had a much greater effect on the microbial community than HA, significantly enriching *Desulfovibrio*, an important metal inactivator (41 times), increasing the concentration of monomethyl arsenate in pore water, and improving the ability of rice to enrich metals. These studies have shown that FA can directly or indirectly contribute to the remediation of metal contaminants by plants.

Microalgae are widely present in ecological environments. New concepts, such as the bacteria–algae system have been applied to the field of water treatment (Jiaqi et al., 2020; Zhigang et al., 2021), and thus the application potential of microalgae in metal bioremediation is gradually being explored (Deng et al., 2020; Jianguo and Xuegang, 2021; Man et al., 2022). Algae widely exist in the natural environment. Their role in the intracellular detoxification mechanism enables them to have high metal accumulation capacities (Oksana et al., 2020; Yan et al., 2022). Microalgae can remove metal contaminants in environmental media through biosorption, bioaccumulation, and biodegradation (Shazia et al., 2021), which have been applied to remove Pb, Cu, Mn, and Zn from wastewater (Lu et al., 2021). FA promotes the growth of algae, enhance the adsorption of algae on metals, improve the growth and metabolism of microalgae, enhance the resistance of algae to metals, and protect microalgae from oxidative damage (Shi et al., 2018; Krachler et al., 2019; Wang et al., 2019).

**Figure 3:**
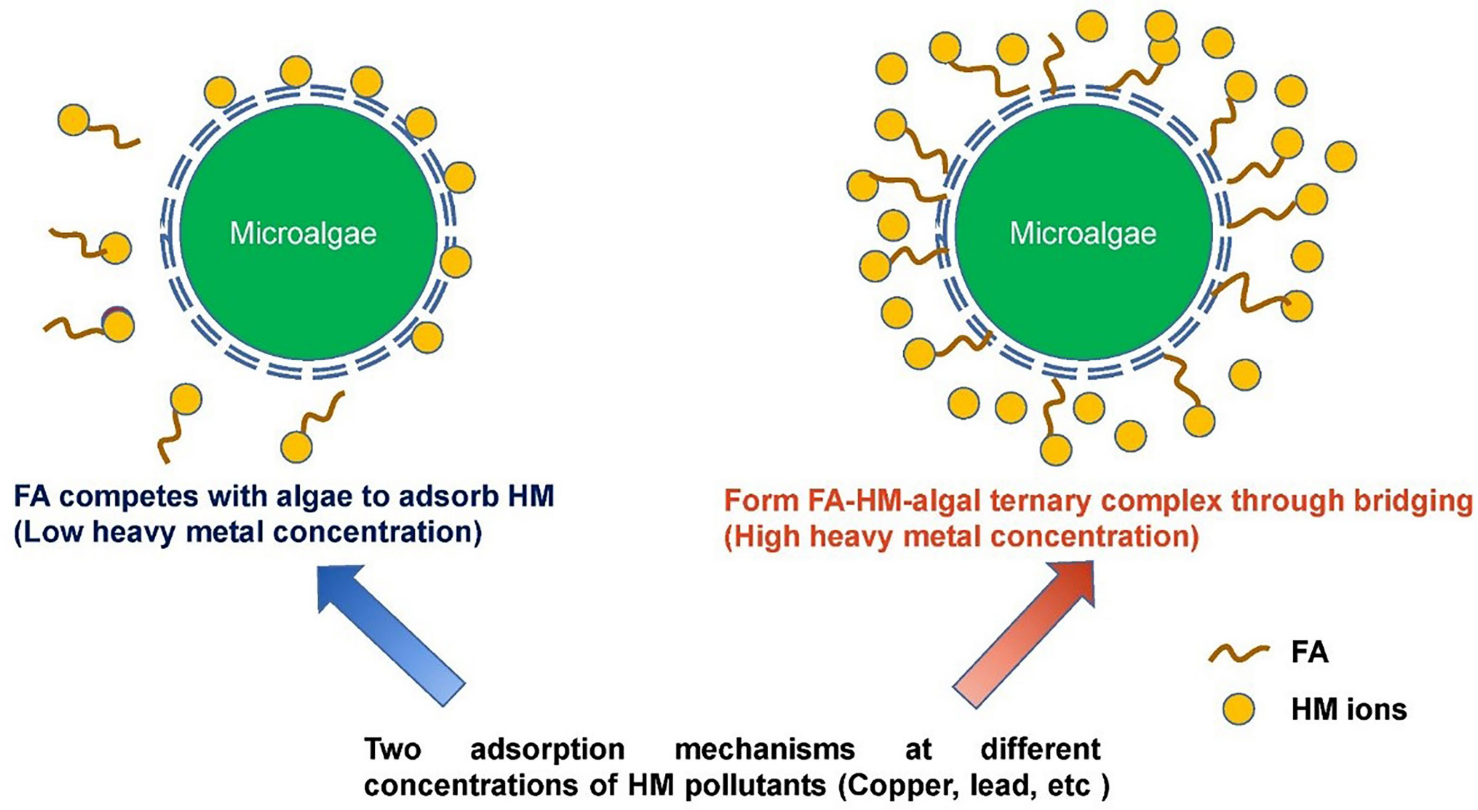
Mechanisms of metal adsorption by microalgae in the presence of FA (Shi et al., 2018).

The mechanism by which FA inhibits the toxicity of metals to algae has been explored. Figure 3 shows the different mechanisms of metal adsorption by microalgae in the presence of FA. Researchers found that FA can effectively improve the concentration of copper in algae and reduce the toxicity of copper to algae; the study showed that the binding mechanism of FA and microalgae changed at different Cu concentrations. FA competes with microalgae for Cu ions to form Cu FA complex when Cu concentration is low, and thus the number of free Cu ions is limited, and the toxicity of Cu ions to algae is reduced. At a high concentration of Cu, FA can bridge with Cu to form ternary complexes of FA, Cu, and algae, which are highly similar to the ternary complexes of Pb, HA, and algae found in previous studies (Cristina et al., 2009; Shi et al., 2018). Lixiao et al. (2017) also found that FA can react with Cd^2+^ through complexation, thus changing the morphology of Cd and thereby significantly improving the absorption of Cd by *microcystis aeruginosa*. Zhang et al. (2022) found that FA can inhibit the toxicity of Ni and Zn to *chlorella pyrenoidosa*; however, FA did not directly participate in the biosorption of metals by algae and only competed with algae by combining with metal ions; this result is different from previous research results on lead in the presence of FA, that is, the formation of ternary complexes on the surface of algae significantly increased algae’s adsorption capacity for lead (the research results showed that the adsorption capacity increased more than 10 times). Given that Pb–FA complexes are mainly limited to the surfaces of algae, the toxicity of Pb on algae can be alleviated (Lamelas et al., 2005; Shi et al., 2020; Weiyan et al., 2020). In conclusion, FA can greatly improve the adsorption capacity and tolerance of algae to Pb, Cu, Ni, and Zn through competition and the formation of FA–metals–algae ternary complex. To date, reports on the use of microalgae in the remediation of metals contaminants in sediments are few probably because the efficiency of FA-assisted microalgal adsorption of metals is susceptible to many factors, such as pH and temperature, during the actual bioremediation of sediments. Nevertheless, the application of microalgae in ecological remediation applications should be enhanced (Bauenova et al., 2021; Ferreira Carraro et al., 2022). Apart from that, studies have shown that DOM generally has a bi-directional effect on the growth of microalgae. Therefore, algae species, environmental factors, and FA dosage should also be taken into consideration when using FA-assisted microalgae for bioremediation in the future (Zheng et al., 2022).

Fulvic acid at an appropriate concentration can enhance the phytoremediation of metal pollutants through multiple actions, but different plant species have diversified demands for FA. Therefore, in practical phytoremediation engineering applications, plant species need to be strictly selected according to local conditions, and aquatic hyperaccumulator plants (such as *Azolla*) are good candidates (Anjuli et al., 2012). Moreover, the bioaccumulation and amplification of metals are subjects of concern. As plant bioavailability of metals increases, it has a high environmental risk as pollution sink, so preventing secondary pollution in practical applications is necessary.

### 3.3. Challenges in remediation technology

Metal contamination in sediments is diverse and geographically specific, so no single remediation technique can be used for all contaminated sediments. In the remediation methods mentioned above, although FA can promote degradation and removal of metal contaminants through adsorption, complexation, and redox reactions, the addition of FA changes environmental pH, redox potential, and porosity. Compared with water and soil environments, the composition of contaminants in sediments is more complex, often accompanied by microplastics and organic pollutants. (Wu et al., 2021) demonstrated that FA and HS inhibit the photoaging of polypropylene microplastics in lake water. In the EKR process, FA may react electrochemically with some organic pollutants and thus produce intermediate products causing secondary pollution. In addition, in future studies, the mechanisms of the joint effects of FA on microbial communities and HM in sediments need to be further elucidated. In particular, the subtle relationship among FA, metals, and microorganisms in alternating wet and dry sediment environments, such as intertidal zones, is worth exploring. Finally, most remediation techniques have side effects to the environment, so the amount of FA used should be further investigated.

## 4. Conclusion and outlook

Fulvic acid is a promising metal remediation tool. As a significant part of DOM, FA can adsorb, complex, and facilitate the oxidation or reduction of many metals and can thus change the morphology, mobility, toxicity, and bioavailability of metals in the natural environment. This article reveals the mechanism of metal remediation using FA and reviews relevant studies on the role played by FA in metal remediation. For physicochemical remediation, FA can enhance the adsorption or passivation of nanomaterials and strengthen the EKR efficiency as a chelating agent. Its light-absorbing active components can generate multifarious reactive oxygen species when exposed to light and are used to promote the photodegradation of organometallic pollutants, such as MeHg. In bioremediation, FA can act as an electron shuttle between microorganisms and as a bridging agent and adsorbent in microalgal and metal systems. FA can also regulate plant gene expression and promote plant growth. This review reveals the potential use of FA in the remediation of metals pollutants in sediment environments, providing a reference for the treatment of metal contaminants.

Although the reaction between FA and metals can increase the efficiency of metal remediation, some aspects still deserve further investigations:

- Sediment environments can be more complex than water and soil environments. The high spatial variability of physicochemical properties (ambient pH, oxygen content, and light intensity) of sediments may have a significant impact on the effectiveness of FA remediation. In addition, as the final destination of all pollutants, the degree of contamination in sediments is greater, and the types of contamination are more complex. The role played by FA in multipollutant systems may be an important subject of interest.
- Fulvic acid usually has a dual role in restoration, as it affects environmental pH, zeta potential, and sediment slumping. Therefore, before the practical application of FA in environmental remediation, its dose, concentration, and molecular weight must be strictly controlled, and its environmental risk must be assessed for the prevention of secondary contamination caused by reagent overdose.
- The abundance and type of functional groups directly determine the adsorption, complexation, and redox ability of FA. As a series of mixtures with similar molecular structures, the structure of FA is extremely complex. Therefore, the adsorption, complexation, and redox capacities of FA from different sources vary greatly. For natural FA, there is a clear regional heterogeneity in its molecular structure; for artificial FA, its molecular structure is closely related to the preparation method (hydrothermal method, composting, etc.). In summary, the source is one of the biggest obstacles to its large-scale practical application. Exploring suitable and stable sources of FA will be the focus of further research.

Although excessive HS may adversely affect the ecological environment, the effect of green and environmentally friendly FA on metal pollution control cannot be ignored. The majority of FA metal remediation research is still conducted in the laboratory, and many research gaps should be addressed before FA can be applied to engineering. Further investigations are necessary to fill these knowledge gaps.

## Author contributions

SS: conceptualization, writing-original draft, and funding acquisition. CS: data curation and formal analysis. JW and ZL: reviewing and editing. YG and WZ: data curation and validation. LZ and YL: supervision and validation. All authors contributed to the article and approved the submitted version.

## Funding

Financial support was provided by National Key R&D Program of China (2022YFE0105600), Natural Science Foundation of Hunan Province (2021JJ40562, 2021JJ40606, 2020JJ4612, and 2020JJ4613), Programs for Science, and Technology Innovation, Department of Transportation of Hunan Province (202034&201802), Hunan key R&D Program Project (2019SK2191), and Natural Science Foundation of Hunan Province (No. 2022JJ40507).

## Conflict of interest

The authors declare that the research was conducted in the absence of any commercial or financial relationships that could be construed as a potential conflict of interest.

## Publisher’s note

All claims expressed in this article are solely those of the authors and do not necessarily represent those of their affiliated organizations, or those of the publisher, the editors and the reviewers. Any product that may be evaluated in this article, or claim that may be made by its manufacturer, is not guaranteed or endorsed by the publisher.
