# Supplementary material for: Applying fulvic acid for sediment metals remediation: Mechanism, factors, and prospect
Source: Front Microbiol. 2023 Jan 9;13:1084097. doi: 10.3389/fmicb.2022.1084097 (PMC9868176; doi:10.3389/fmicb.2022.1084097)
Supplement: Supplementary file 1 [file Image_1.pdf]

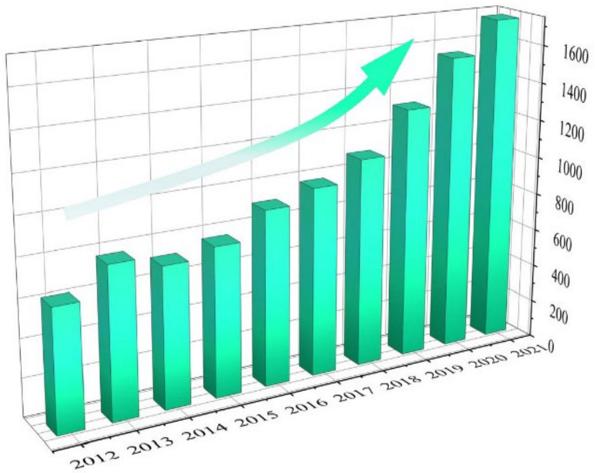

Supplementary Figure 1. Trend chart of the number of publications on FA in the past decade

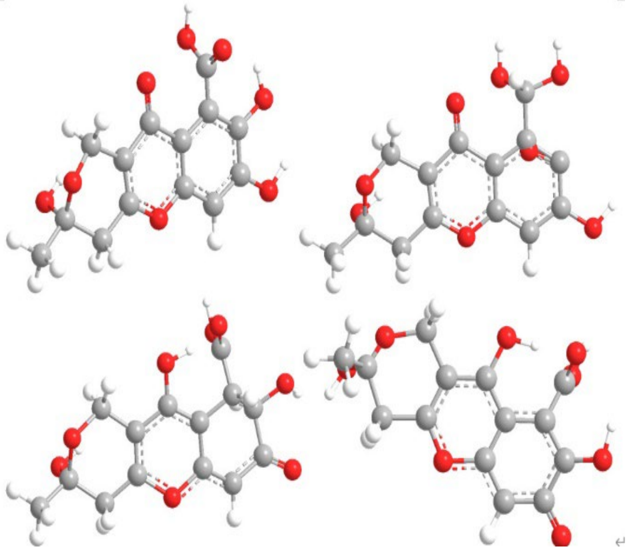

Supplementary Figure 2. Possible three-dimensional diagrams of a class of FA (Grey, red, and white spheres represent C, O, and H atoms respectively) (Li et al., 2018).
